# The Integration of Meditation and Positive Psychology Practices to Relieve Stress in Women Workers (Flourish): Effects in Two Pilot Studies

**DOI:** 10.3390/bs11040043

**Published:** 2021-03-26

**Authors:** Fabio R. M. dos Santos, Shirley S. Lacerda, Cassia C. Coelhoso, Carla R. Barrichello, Patricia R. Tobo, Elisa H. Kozasa

**Affiliations:** 1 Hospital Israelita Albert Einstein, São Paulo CEP 05601-901, Brazil; fabiomunhoz@hotmail.com (F.R.M.d.S.); lacerdass@gmail.com (S.S.L.); cassiaccoelhoso@gmail.com (C.C.C.); 2 Natura Cosméticos, Cajamar CEP 07790-190, Brazil; carlabarrichello@natura.net (C.R.B.); patriciatobo@natura.net (P.R.T.)

**Keywords:** meditation, mindfulness, positive psychology, stress, intervention

## Abstract

Meditation and positive psychology have been reported as promising approaches to deal with stress. This exploratory pilot study aims to evaluate the efficacy of meditation and positive psychology-based intervention on stress. Two experiments are reported; in the first one, 29 healthcare professionals were included (intervention = 14; control = 15), and the second one included 57 women managers (intervention = 27; control = 30). All participants were evaluated before and after eight weeks for levels of stress, anxiety, depression, and psychiatric symptoms. In Experiment 1, intervention group participants showed a reduction in stress levels and anxiety symptoms, and in Experiment 2, participants in the intervention group showed a decrease in stress, psychiatric symptoms, and sleep quality, when compared to the control group. A multi-component intervention based on positive psychology and meditation practices was effective at reducing stress as well as improving related stress parameters such as anxiety, psychiatric symptoms, and sleep quality.

## 1. Introduction

The Stress Survey in America, an American Psychological Association report, pointed out that work is a major source of stress for 61% of Americans [1]. The negative effects of stress occur at both personal and organizational levels. Stress-related disorders may lead to mental disorders as well as cardiovascular problems [2].

There is considerable evidence that the stress inherent to professional activity affects healthcare workers, a population considered particularly vulnerable to its effects. It is well known that stress can lead to psychological distress and is related to the onset of psychiatric disorders in healthcare professionals [3]. Stress may also reduce professional effectiveness as it decreases attention and impinges on decision-making skills [4]. Stress may also lead to burnout syndrome, which can manifest itself as emotional exhaustion, depersonalization, and a sense of low personal accomplishment [5]. Healthcare workers’ burnout has been significantly associated with self-reported deficient patient care [6]. The stress experienced by emergency nurses may also result in absenteeism, lower productivity, and lack of job satisfaction [7].

In addition, management positions are also considered as high-stress ones because of the intense workload, sometimes demanding 60-h work weeks, and because of the psychological pressure to accomplish all tasks required [8]. Higher positions in companies entail higher stress levels [9]. Additionally, it is considered that women in leadership positions would be more susceptible than men to emotional stress or fatigue, probably due to excessive empathy [10].

There is plenty of evidence that stress affects managers and healthcare professionals, and therefore, there is great need for interventions aimed to relieve stress and foster well-being in these populations. 

Some widely validated programs in stress management are based on Mindfulness Meditation practices. According to Kabat-Zinn, mindfulness can be defined as “the awareness that emerges through paying attention, on purpose, in the present moment, and non-judgmentally to the unfolding of experience moment by moment” [11]. Some studies have investigated the effects of mindfulness practices on healthcare professionals, and results from a systematic review suggest that mindfulness practices have the potential to decrease stress among these professionals [12]. A study conducted in the Brazilian population evaluated a mindfulness-based stress reduction program, which was adapted to the time constraints of a business environment and was effective in reducing stress as well as depression, anxiety, and non-severe psychiatric symptoms. Improvement of attention skills and of mental processing speed were also observed [13]. A review of empirical studies examined the potential benefits of mindfulness programs aimed at enhancing well-being and coping with stress; the observations indicated benefits in the domains of physical and mental health [14].

Among the meditative practices described and evaluated in academic publications, research has started to explore loving-kindness meditation (LKM), a traditional Buddhist meditation commonly practiced in the context of mindfulness. LKM involves attitudes of unconditional love, kindness and compassion for oneself and others. The existing reports suggest that LKM is associated with an increase in positive effect and a decrease in negative emotions [15]. Mindfulness and loving-kindness meditation can increase self-compassion and well-being in healthcare professionals, and reduce empathetic distress, fatigue, and burnout [16]. A Brazilian study, conducted with nurses working in a hospital, evaluated the effects of a mindfulness and loving-kindness meditation program and reported significant reduction in perceived stress scores and in burnout, depression, and anxiety symptoms [17].

The existing evidence shows that mental healthcare systems have traditionally focused more on treatment of mental disorders than on prevention, being basically focused on reducing patients’ symptoms or deficits and less concerned with promoting positive aspects. Positive psychology is the scientific study of happiness and human potential. During the last decade, positive psychology has broadened psychology’s focus towards a more optimistic aspect of personality. Seligman and Csikszentmihaly pioneered the base of positive psychology in their article ‘Positive psychology: An introduction’, published in an issue of *American Psychologist* [18]. Seligman defines positive psychology as the study of humans’ positive side aimed at developing personal strengths and virtues as well as optimal functioning and well-being [19]. It does not mean that a positive psychology approach bypasses or neglects negative aspects of disease. Positive psychology offers a novel paradigm to mental and emotional health, as it seeks to promote the development of virtues, quality of life and well-being, instead of just treating a patient’s disease [20].

Positive Psychology Intervention (PPI) is an intervention in tune with the theoretical principles of positive psychology, (i.e., training, exercise, therapy) aimed at augmenting positive feelings, cognitions, or behaviors [21]. Positive psychology interventions seem to be promising techniques to improve employee well-being and performance [22]. There are some published studies on PPI with significant results in the promotion of well-being and reduction of depressive symptoms. Among them are practices of gratitude, identification and use of strengths and virtues, all aiming to evoke positive emotions and well-being [20,21,22]. A meta-analysis of 39 randomized studies concluded that positive interventions can be effective at promoting wellness parameters, as well as in assisting to decrease adverse psychological symptoms. Various intervention practices were reported, such as writing about positive experiences, maintaining a gratitude diary, counting one’s blessings, and savoring the moment [23]. 

In recent years, some multi-component interventions integrating meditation and positive psychology have been described and evaluated in literature. Recently, a meta-analysis investigated the effectiveness of multi-component positive interventions [24]. The authors found evidence that this type of intervention improved mental health and suggested that further well-conducted research of diverse populations is needed to support claims of its efficacy. According to Kabat-Zinn, mindfulness can be defined as ‘‘the awareness that emerges through paying attention, on purpose, in the present moment, and non-judgmentally to the unfolding of experience moment by moment” [11].

Some publications have pointed to the promising possibilities of approaches integrating the fields of meditation and positive psychology [25]. This novel consideration proposes that meditative practices and positive psychology interventions can influence and enhance one another, conducing to well-being. In addition, meditative practices can, by themselves, promote virtues that are pertinent to the study of positive psychology, like perspective view, gratitude, curiosity, or self-control [26,27]. 

Some meditation-based programs to promote compassion and self-compassion which include appreciation and gratitude practices at some stage are also described in the academic literature. We can find these practices inserted in programs like Compassion Cultivation Training [28], Cognitive-Based Compassion Training [29], and Mindful Self-Compassion Program [30]. 

As mentioned, there is significant published evidence that profession-related stress negatively impacts healthcare professionals, leading to increased depression, decreased job satisfaction, and psychological distress. To this date, there are no published academic studies on combined meditation and positive psychology interventions specifically aimed at relieving stress in women workers. To address this point, the current study examined the effects of a novel intervention model that integrates elements of meditation with elements of positive psychology interventions; we call this program “Flourish”. The present pilot study evaluates the effect of this model in two experiments: one was carried out on a group of healthcare professionals and the other on women in management positions.

The Flourish program is a multi-component mindfulness and positive psychology-based intervention aimed to enhance well-being. This protocol proposes a well-being program based on meditation and positive psychology principles such as human development, stimulation of virtues, and improvement of quality of life and well-being. The investigators’ hypothesis is that this program may reduce stress-related problems in the participants. This program was also adapted into an app. It was evaluated for its efficacy at promoting stress management and well-being among working women. The results showed that the Flourish app was effective in reducing employees’ stress and at improving well-being [31].

To assess the effects of a multi-component meditation and positive psychology-based intervention in reducing stress, we conducted two pilot experiments; the first, on a group of women working as healthcare professionals and the second on a group of women in management positions.

## 2. Materials and Methods

### 2.1. Experiment 1

#### 2.1.1. Participants

An invitation to healthcare women workers interested in participating in the Flourish Program was divulged via intranet at Hospital Israelita Albert Einstein (HIAE), Brazil. In response to the invitation, 141 women showed interest. Among those interested, 52 signed the informed consent form and entered the study. The group included nurses (73%), pharmaceutical workers (15%), physicians (6%) and administrative workers (6%). The study was approved by the ethical committee of HIAE. The inclusion criteria were being a woman and a healthcare worker. The exclusion criteria were having experienced previous psychiatric or neurological disorders and/or being under psychological or psychiatric treatment.

#### 2.1.2. Design

Participants were allocated in equal numbers to the intervention group and to the control group. Due to individual working hours and the availability of intervention classes, it was not possible to perform randomization. Therefore, participants were allocated to the groups according to their working schedules. The intervention group participated in the Flourish Program for 8 weeks. Twenty-two original participants either abandoned or were excluded from the study, and the remaining 30 participants completed the program. The participants were evaluated at two moments: pre- and post-intervention. After the final evaluation, participation in the Flourish Program was offered to control group participants.

The evaluation instruments were applied as a baseline (pre-intervention) and after 8 weeks (post-intervention). After the baseline evaluation, the intervention group participated in the Flourish Program for 8 weeks, while control group participants were assigned to a waiting list. After 8 weeks, a second evaluation was performed. Finally, the control group was admitted to the intervention program (Flourish Program).

### 2.2. Experiment 2

#### 2.2.1. Participants

Ninety-nine women with complaints of stress, occupying management positions at administrative and sales departments of a multinational cosmetics company were recruited. The participants’ ages were between 25 and 60 years, and their education level was at least a university bachelor’s degree. All were willing to participate in the controlled trial of the well-being Flourish program.

#### 2.2.2. Design

Initially, the participants were randomly allocated to the waiting list group (control) or to the intervention group (Flourish Program). It was later necessary to reallocate several participants to intervention classes that fit their busy work agendas (particularly sales managers, who had seasonal workflow bottlenecks). We excluded from the study seven volunteers with mental health issues (verified by an experienced physician). Thirty-four women were not able to adjust their routine tasks to the activities or tasks proposed by the program. Eventually, 58 women were included in the study.

The experiments reported herein are part of a broader project registered with Clinical Trials (NCT02164188), which includes measurement of other variables as well as testing an applicative version of the same program.

### 2.3. Intervention (The Same Protocol Was Applied in Experiments 1 and 2)

The Flourish Program is a multi-component intervention based on meditation practices and positive psychology principles, intended to promote well-being. The format is one-hour-and-a-half meetings for eight weeks, accompanied by daily practices. Every meeting started with a short interactive session in which the participants commented on how they had carried out the program-related practices during the week. This was followed by a short period of meditative practice. Following, the chairperson presented the week’s theme and the participants offered their comments or questions. At the end of each meeting, the participants engaged in meditation practices. All participants received a set of four audios as guidance for their daily meditation practices for the duration of the program. The content topics in the audios were body scanning, paying attention to breathing (mindful breathing), empathetic joy meditation and loving-kindness meditation. Data from these experiments were collected in 2015.

### 2.4. Meeting Topics and Activities in the Flourish Program

- 1st Week—Introducing the program. Theory: what is attention and focus. Practices: gratitude diary; counting blessings or reflecting on those aspects of one’s life for which one should be grateful.
- 2nd week—Theory: life is relationship, interdependence and a sense of belonging. Practices: sharing or talking about the gratitude diary, body scanning, mindful attention focused on corporeal experience.
- 3rd week—Theory: cultivating positive emotions and empathy. Practices: awareness of the breathing act (mindful breathing) and body scanning.
- 4th week—Theory: cultivating compassion. Practice: loving-kindness meditation and mindful breathing.
- 5th week—Theory: gratitude, reflecting on personal life aspects for which to be grateful. Practice: sharing the gratitude diary and empathetic joy meditation.
- 6th week—Theory: PERMA model (positive emotion, engagement, meaning, positive relationships and achievement). Practices: mindful breathing, loving-kindness meditation and sharing the gratitude diary.
- 7th week—Theory: character strengths: identifying and using them. Practices: sharing character strengths; empathetic joy meditation.
- 8th week—Theory: hope, expectations in life. Review of the contents and carried-out practices. Practice: mindful breathing.

### 2.5. Instruments (The Same Were Used for Both Experiments)

Perceived Stress Scale (PSS): This is a 14-item instrument rated on a 4-point Likert scale (almost never to always), which evaluates the perception of stressful events [32]. An example item is, “In the last month, how often have you felt nervous and stressed?” We added the scores of all questions to calculate total scores, inversely calculating items with positive connotation against stress (0  =  4, 1  =  3, 2  =  2, 3  =  1, 4  =  0). The PSS showed adequate internal consistency (α  =  0.82) [33].

Self-Report Questionnaire-20 (SRQ-20): An inventory for the detection of psychiatric symptoms, with 20 questions about mental health. The cut-off value was 7/8, with 86.33% sensitivity and 89.31% specificity [34]. The score range is 0–20.

Beck Depression Inventory (BDI): The BDI includes 21 items describing depression symptoms, each item rated on a scale from 0 to 3. The total score range is 0–63 and the cut-off value to discriminate mild to moderate symptoms of depression was 20 points, with 0.77 sensitivity and 0.95 specificity [35].

Beck Anxiety Inventory (BAI): The BAI is comprised of 21 items describing anxiety symptoms, each item rated on a scale from 0 to 3 and whose total score range is 0–63. The internal consistency was 0.91 and the test–retest reliability was 0.99 for a sample of the Brazilian population [36].

Pittsburgh Sleep Quality Index (PSQI): The PSQI assesses sleep quality in the last month by means of 19 items that comprise seven components. These components are subjective sleep quality, sleep latency, sleep duration, habitual sleep efficiency, sleep disturbance, use of sleeping medication, and daytime dysfunction. Each component is weighted on a 0–3 scale, and then the seven components are summed to yield a global score that ranges from 0 to 21 [37]. 

### 2.6. Statistical Analysis 

Descriptive statistics were computed for study variables. To check for preexisting group differences at study entry despite randomization, Student’s *t* test for continuous variables and chi-square analyses for categorical variables were conducted on descriptive variables and outcome measures.

Data are presented as mean and standard deviation. In inferential statistics—after a study of presumptions and covariate variables—a repeated measures analysis of variances was used (repeated measures ANOVA). In Experiment 2, due to the significant difference in age between the intervention and control groups, the repeated measures ANOVA was performed covariate by age. The analysis was performed using the JASP program (Version 0.13; JASP Team; 2020). These statistical methods were applied to both experiments.

## 3. Results

### 3.1. Experiment 1

**Figure 1:**
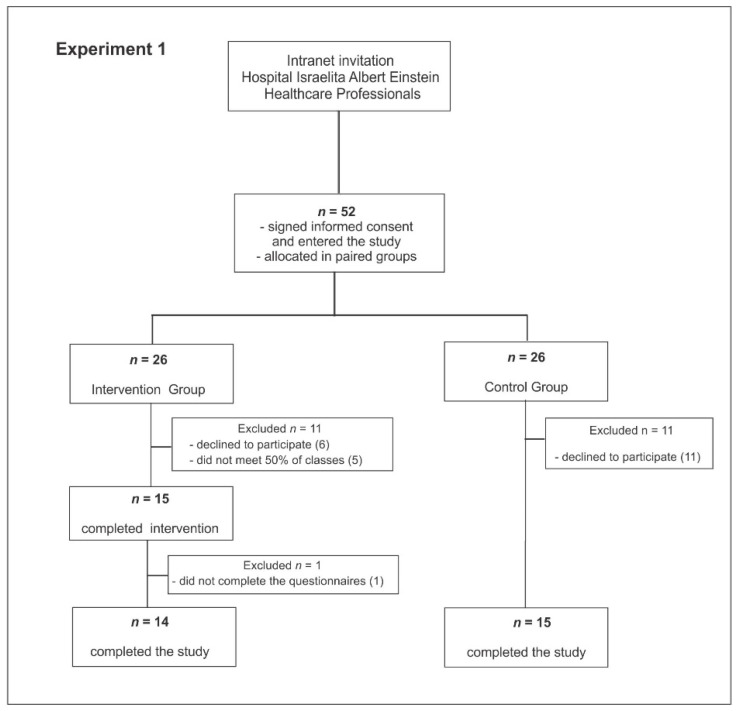
Consort flow chart (Experiment 1).

One hundred and forty-one women contacted the researchers showing interest in participating. Of those who did, 89 were excluded for the following reasons: not meeting inclusion criteria—were not healthcare professionals (*n* = 25); declined for not having time to follow the program (*n* = 48); and failure to attend the scheduled activities (*n* = 16). A reason for the high number of exclusions was an outbreak of H1N1 influenza in the city, which demanded extra work from healthcare professionals, preventing attendance to the program sessions. Fifty-two women signed the informed consent form, enrolled in the experiment, and were allocated to paired groups. Twenty-three participants abandoned the program or were lost before evaluation. Another 17 participants further declined to participate, and five did not attend 50% of the meetings. The participants who completed the study were 29, 14 belonging to the intervention group and 15 to the control group (Figure 1).

#### 3.1.1. Baseline Characteristics

Baseline characteristics of Experiment 1 are summarized in Table 1. Intervention and Control Group were similar in all evaluated variables. There were no statistically significant differences in outcome variables between groups at baseline: perceived stress, anxiety, psychiatric symptoms (Self-Report Questionnaire), depression, sleep quality, mindful awareness attention and self-compassion. This analysis was performed after the intervention was completed, with 14 and 15 subjects in the intervention and control group, respectively (Table 1).

#### 3.1.2. Changes in Perceived Stress after the Intervention

In the experiment with healthcare professionals, the repeated measures ANOVA test was conducted to test the effect of the intervention on variables over time and the results indicated that the intervention decreased perceived stress over time. Table 2 shows that the effect of the intervention on perceived stress was significant when comparing intervention and control groups (F_1_ = 4.86; *p* = 0.036). A post-hoc test shows that the difference is between baseline and after 8 weeks in the intervention group (t_1_ = 5.28; *p* < 0.001).

#### 3.1.3. Changes in Other Variables Studied after the Intervention

The repeated measures ANOVA test also indicated that the intervention decreased anxiety symptoms (F_1_ = 5.67; *p* = 0.025) over time. There were no significant differences between groups concerning depression, psychiatric symptoms and sleep quality (Table 2). A post-hoc test shows that the difference is between baseline and after 8 weeks in the intervention group (t_1_ = 2.83; *p* < 0.034).

### 3.2. Experiment 2

**Figure 2:**
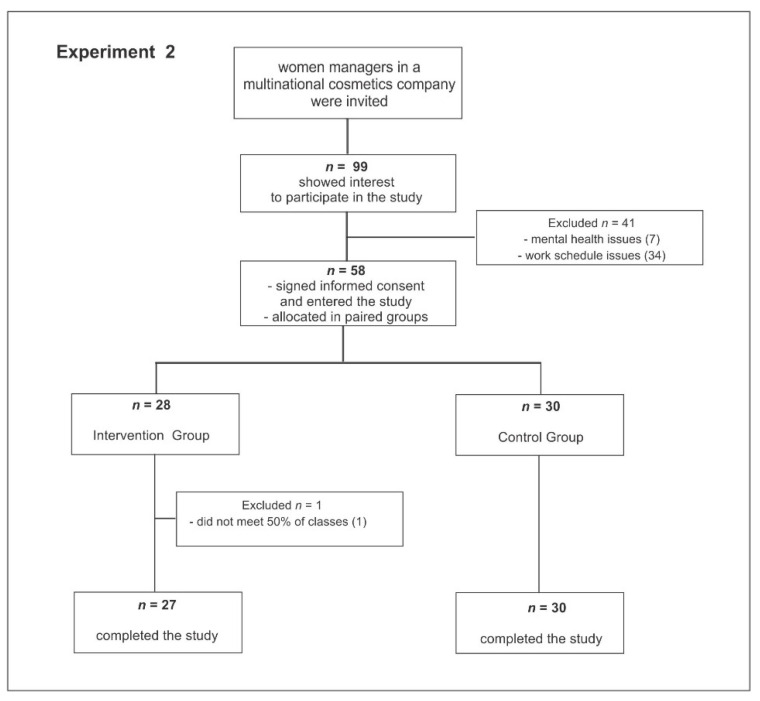
Consort flow chart (Experiment 2).

Women occupying management positions in a multinational company were invited via intranet to participate in the Flourish program. In response to the invitation, 99 women expressed interest in participating in the study. Among those interested, 58 signed the informed consent form and entered the study; 28 in the intervention group and 30 in the control group. One participant in the intervention group was excluded because she failed to attend 50% of classes. Fifty-seven participants completed the study, 27 in the intervention group and 30 in the control group (Figure 2). Most participants were Caucasian, married, Catholic, and there were no differences between intervention and control groups concerning these variables. The average age of the control group and intervention group was 42.4 (SD 8.37) and 46.64 (SD 6.93), respectively; the difference was significant at *p* < 0.05. Therefore, the statistical analysis was adjusted to this variable.

#### 3.2.1. Baseline Characteristics

The age difference between the control and the intervention group in Experiment 2 was statistically significant. Therefore, we adjusted the statistical analysis to this variable (Table 3). Most of the participants were married (60% in control group and 61.7% in intervention group), Catholic (51.8% in control and 46.7% in intervention group) and there were no differences between groups concerning these variables. There were no significant differences between groups in the scores of the psychometric variables at baseline (Table 3).

#### 3.2.2. Changes in Perceived Stress after the Intervention

The repeated measures ANOVA test was conducted to test the effect of the intervention on variables over time and the results, as shown on Table 4, indicate that the intervention decreased perceived stress over time in comparison to the control group. The parameter perceived stress (PSS) was significantly different between the intervention and control groups (F_1_ = 8.89; *p* = 0.019). A post-hoc test shows that the difference is between baseline and after 8 weeks in the intervention group (t_1_ = 2.83; *p* = 0.039).

#### 3.2.3. Changes in Other Variables Studied after the Intervention

The repeated measures ANOVA test also indicated that the intervention reduced psychiatric symptoms (F_1_ = 7.87; *p* = 0.007) and improved sleep quality (F_1_ = 5.89; *p* = 0.019) over time. A post-hoc test shows that for psychiatric symptoms (t_1_ = 3.09; *p* = 0.019) as well as for sleep quality (t_1_ = 2.97; *p* = 0.027) the differences were between baseline and after 8 weeks in the intervention group. There were no significant differences between groups related to depression and anxiety symptoms (Table 4).

## 4. Discussion

This study aimed to evaluate the effects of an 8-week meditation and positive psychology-based intervention (Flourish Program) on the stress perceived by women working as healthcare professionals or in management administrative positions. In addition, we analyzed the effects of the program on psychiatric symptoms, anxiety, depression, sleep quality, mindful awareness, and self-compassion. The experiments confirmed, for both groups of workers, that the program was effective at reducing the participants’ stress-related problems measured through the Perceived Stress Scale.

In Experiment 1, the control and intervention groups had similar baseline profiles, enabling the conclusion that differences in the analyzed parameters appearing after intervention could be attributed to the program. Healthcare professionals who attended the intervention program reported significant reduction of both perceived stress (F_1_ = 4.86; *p* = 0.036) and anxiety symptoms (F_1_ = 5.67; *p* = 0.025). In Experiment 2, women in management positions reported significant reduction of perceived stress (F_1_ = 8.89; *p* = 0.019) and psychiatric symptoms (F_1_ = 7.87; *p* = 0.007) as well as improvement in sleep quality (F_1_ = 5.89; *p* = 0.019) in comparison to the respective control group.

There is plenty of evidence that women—in both types of occupation, healthcare and management—are exposed to high levels of stress that may result in burnout, reduced performance, absenteeism, and high personnel turnover [38,39]. Additionally, women are considered more susceptible than men to emotional stress and fatigue, probably because they are more empathic [10]. Awareness of these problems generates the need for effective interventions to relieve the harmful effects of stress. Within this context, the present study contributes to meet this demand by evaluating a multi-component intervention aimed at promoting well-being and at relieving stress.

Diverse types of interventions to reduce stress at work have been suggested and tested. A published systematic review on the effectiveness of such interventions among healthcare workers underscored the need for more active stress management policies by institutions and companies, large-scale implementation, and better-quality trials [39].

Meditation-based interventions are effective at decreasing stress in healthcare professionals, both in nurses [40], and physicians [41]. A systematic review investigated the efficacy of mindfulness-based interventions at relieving stress among healthcare professionals [12]. It was concluded that this approach has the potential to reduce stress although more high-quality research is needed. For us, those studies indicate that meditative practices should be considered when developing interventions to relieve stress and promote well-being. Our program is therefore aligned with this approach, incorporating practices such as mindful breathing, body scanning, and mindful attention focused on bodily experience.

On the other hand, interventions based on positive psychology are also effective in promoting well-being and reducing stress [42,43]. One of the well-validated practices in positive psychology is gratitude. An effective strategy to enhance well-being is to persuade people to identify their blessings or to reflect on those aspects of their lives for which they are grateful. Such a strategy has the potential to improve well-being by fostering gratitude through simple exercises such as keeping a gratitude diary [44,45]. In our study, we used the gratitude diary together with meditation practices and the results show that integrating these practices can be effective at reducing stress levels in female professionals. The potential of combining these two approaches was recognized as meditation and positive psychology practices together offered better gains in well-being than either practice intervention offered alone [25]. The integrated use of gratitude and meditative practices can also be identified in programs such as Cognitive-Based Compassion Training (CBCT) [29] and Compassion Cultivation Training (CCT) [28]. These programs include a module for appreciation and gratitude, focused mainly on people who provide us with good things. For example, while keeping a diary of gratitude we may write that we are grateful for a piece of clothing, whereas in the CBCT and CCT programs, the focus is to thank the people who contributed to make the clothing available; those who planted, transported, prepared, etc.

Regarding the use of strengths and virtues—although both types of interventions, mindfulness and use of strengths and virtues, fosters well-being individually—only few studies started to investigate their potential overlap and synergistic effect. In a recent study, Pang and Ruch evaluated a program that integrates mindfulness and character strengths, named “mindfulness-based strengths practice” (MBSP), and compared it to the “mindfulness-based stress reduction” (MBSR). The study examined the effectiveness of training regarding well-being and work-related outcomes. The findings suggest that mindfulness alone functions better with well-being, whereas the combined approach seems to influence motivation, and thus bolsters task performance [46]. The present study used a similar approach in an intervention by integrating meditation practices and identification and use of strengths and virtues.

Some limitations must be pointed out in both experiments. First, that the control and intervention groups were allocated by convenience. The non-randomization was due to the professional schedules of the participants in relation to the availability of the intervention classes. Another limitation is the lack of a placebo group that could assess the effect of intervention expectation. The participation in group-based courses may promote a sense of social belonging and could thereby alleviate the symptoms of stress in participants. Future studies may bring important contributions by using active control groups with similar face-to-face meetings. Long-term outcome measures are recommended, with a longer follow-up assessment. We suggest for future studies to evaluate the application of this program in male populations to investigate the effectiveness of this type of intervention on those subjects. We also suggest that future studies compare PPI and meditation separately. For example, a methodological design with four groups would be very promising: control; PPI only; meditation only; and meditation and positive psychology-based intervention.

## 5. Conclusions

A multi-component meditation and positive psychology-based intervention was effective at reducing stress and anxiety symptoms in female healthcare professionals and managers. Meditation and positive psychology practices can be successfully integrated in interventions aimed to reduce stress and promote well-being.

## Figures and Tables

**Table 1:** Experiment 1: Demographic characteristics at baseline of intervention and control group.

	Control Group (*n* = 15)	Intervention Group (*n* = 14)	Value (df)	*p* Value
	Mean (SD)/*n* (%)	Mean (SD)/*n* (%)		
Age	36.07 (7.60)	40.20 (6.74)	−1.58 (27)	0.126
Marital status				
Single	2 (13.33%)	5 (35.71%)	3.18 (4)	0.528 ^c^
Married	8 (53.34%)	5 (35.71%)		
Partnered	2 (13.33%)	2 (14.29%)		
Divorced	2 (13.33%)	2 (14.29%)		
Other	1 (6.67%)	0 (0%)		
Religion				
No religion	4 (26.67%)	2 (14.29%)	9.11 (4)	0.058 ^c^
Catholic	5 (33.33%)	4 (28.57%)		
Evangelical	5 (33.33%)	1 (7.14%)		
Spiritist	1 (6.67%)	5 (35.71%)		
Other	0 (0%)	2 (14.29%)		
PSS	23.20 (4.36)	26.43 (5.64)	−1.01 (27)	0.320 ^t^
SRQ	9.27 (3.86)	10.86 (2.90)	−0.73 (27)	0.470 ^t^
BDI	19.27 (9.84)	20.50 (8.05)	0.04 (27)	0.970 ^t^
BAI	11.93 (7.00)	17.93 (8.04)	−1.74 (27)	0.093 ^t^
PSQI	8.93 (3.28)	9.00 (3.40)	0.31 (27)	0.758 ^t^

Legend: ^t^ = Student t test; ^c^ = Chi-squared test; n = Number of participants; SD = Standard Deviation; PSS = Perceived Stress Scale; SRQ = Self-Report Questionnaire; BDI = Beck Depression Inventory; BAI = Beck Anxiety Inventory; PSQI = Pittsburgh Sleep Quality Index.

**Table 2:** Experiment 1: Comparison between intervention and control groups at baseline and after 8 weeks.

	Control Group (*n* = 15)		Intervention Group (*n* = 14)		Time Effect	Group Effect	Time* Group Effect
	Baseline	After 8 Weeks	Baseline	After 8 Weeks			
	Mean (SD)	Mean (SD)	Mean (SD)	Mean (SD)			
PSS	23.20 (4.36)	22.13 (7.92)	26.43 (5.64)	21.07 (5.50)	0.003 **	0.594	0.036 *
SRQ	9.27 (3.86)	9.40 (4.40)	10.86 (2.90)	8.50 (5.18)	0.077	0.811	0.050
BDI	19.27 (9.84)	15.73 (10.27)	20.50 (8.05)	15.07 (11.47)	0.016 *	0.931	0.591
BAI	11.93 (7.00)	12.87 (9.47)	17.93 (8.04)	12.43 (7.61)	0.102	0.310	0.025 *
PSQI	8.93 (3.28)	6.40 (3.87)	9.00 (3.40)	7.29 (3.97)	0.002 **	0.696	0.510

Legend: * = *p* < 0.05; ** = *p* < 0.01; *n* = Number of participants; SD = Standard Deviation; PSS = Perceived Stress Scale; SRQ = Self-Report Questionnaire; BDI = Beck Depression Inventory; BAI = Beck Anxiety Inventory; PSQI = Pittsburgh Sleep Quality Index.

**Table 3:** Experiment 2: Demographic characteristics at baseline of intervention and control groups.

	Control Group (*n* = 30)	Intervention Group (*n* = 27)	Value (df)	*p* Value
	Mean (SD)/*n* (%)	Mean (SD)/*n* (%)		
Age	40.93 (8.17)	46.07 (7.87)	−2.46 (56)	0.017 ^t^
Marital status				
Single	2 (6.68%)	3 (10.71%)	2.16 (3)	0.539 ^c^
Married	16 (53.32%)	16 (60.71%)		
Partnered	6 (20.00%)	2 (7.14%)		
Divorced	6 (20.00%)	6 (21.43%)		
Religion				
No religion	8 (26.68%)	4 (14.81%)	3.62 (5)	0.604 ^c^
Catholic	14 (46.68%)	14 (51.85%)		
Protestant	0 (0%)	1 (3.70%)		
Evangelical	1 (3.33%)	3 (11.11%)		
Spiritist	4 (13.33%)	4 (14.81%)		
Other	3 (10.00%)	1 (3.72%)		
PSS	21.03 (7.04)	19.82 (7.45)	0.64 (56)	0.527 ^t^
SRQ	6.00 (3.50)	6.35 (3.88)	−0.37 (56)	0.714 ^t^
BDI	12.60 (6.45)	13.57 (9.26)	−0.47 (56)	0.643 ^t^
BAI	9.17 (6.68)	9.18 (6.57)	−0.01 (56)	0.995 ^t^
PSQI	7.17 (3.19)	7.96 (4.17)	−0.79 (56)	0.431 ^t^

Legend: ^t^ = Student t test; ^c^ = Chi-squared test; n = Number of participants; SD = Standard Deviation; PSS = Perceived Stress Scale; SRQ = Self-Report Questionnaire; BDI = Beck Depression Inventory; BAI = Beck Anxiety Inventory; PSQI = Pittsburgh Sleep Quality Index.

**Table 4:** Experiment 2: Comparison between the control and the intervention groups at baseline and after 8 weeks (covariate by age).

	Control Group (*n* = 30)		Intervention Group (*n* = 27)		Time Effect	Group Effect	Time* Group Effect
	Baseline	After 8 Weeks	Baseline	After 8 Weeks			
	Mean (SD)	Mean (SD)	Mean (SD)	Mean (SD)			
PSS	21.03 (7.04)	21.46 (6.10)	19.82 (7.45)	17.39 (8.14)	0.369	0.093	0.019 *
SRQ	6.00 (3.50)	6.33 (4.35)	6.36 (3.89)	4.93 (4.43)	0.173	0.355	0.007 *
BDI	12.60 (6.45)	11.73 (6.68)	13.57 (9.26)	10.68 (11.33)	0.229	0.466	0.161
BAI	9.17 (6.68)	8.43 (7.71)	9.18 (6.57)	7.92 (7.42)	0.418	0.465	0.980
PSQI	7.17 (3.19)	7.34 (3.54)	7.96 (4.17)	6.35 (3.91)	0.294	0.257	0.019 *

Legend: * = *p* < 0.05; *n* = Number of participants; SD = Standard Deviation; PSS = Perceived Stress Scale; SRQ = Self-Report Questionnaire; BDI = Beck Depression Inventory; BAI = Beck Anxiety Inventory; PSQI = Pittsburgh Sleep Quality Index.

## Data Availability

The authors will share data from the study upon reasonable request to the corresponding author.
